# Prevention of Wastage in Convalescent Depots

**Published:** 1918-09

**Authors:** C. G. Brown


**Prevention of Wastage in Convalescent Depots.** Lt. Colonel C. G. Brown, R. A. M. C.

Colonel Brown said he would approach the subject from the point of view of the commanding officer of a convalescent depot.

What strikes one first is that even during periods of heavy fighting the *proportion of sick to wounded* passing through convalescent depots is very high. In the speaker's own depot, during the period January 1st to July 16th, 1918, there were 21557 admissions. Of these 7004 were due to wounds and 14553 were due to sickness. At the present time the ratio is 5 to 1, and the average over a period of 12 months would be about 3 to 1. Amongst the admissions due to sickness, 3354 are due to P. U. O., 2041 to I. C. T., 1467 to influenza, 583 to trench fever, 222 to boils, 165 to scabies and 76 to impetigo — a total of 7908. The speaker has selected these diseases as having for their origin our persistent enemy the louse or want of personal hygiene. In view of recent work and allowing for errors of diagnosis, he thinks it may be suggested that the vast majority of the P. U. O. and influenzas are in reality trench fevers and due to the *louse virus*. He is prepared for any pathologist present to dispute this. The figure for admissions due to these diseases is half that for the total admissions for sickness and actually exceeds that for the total admissions for wounds. The speaker declared, therefore, that the principal cause of our sick wastage from the front areas is the louse. To attack and if possible abolish the louse would result in no more overcrowding and congestion of medical units and transportation.

A second source of loss of man-power from the armies is found among the class of men whom the speaker likened to the traveling *circus*. There is undoubtedly a large proportion of men who spend their whole lives in circulating between hospitals and convalescent depots on the L. of C. This type of man knows all the best cards to play and how to play them. He is an adept at obtaining employment and so prolonging his stay in a convalescent depot.

The speaker gave in detail the amusing histories of two cases of clever evasion and " camouflage " whereby the offenders succeeded in putting in their time anywhere but with their units.

Another cause of wastage is the *enlistment* and reenlistment *of unsuitable men.* The speaker read the following significant letter from a neurasthenic:

"Sir,

"This is some of my history since 1915. In 1915 I enlisted in the O. T. C. They only kept me about three days and rejected me. I then went on war work but again in 1916 I enlisted in the R. F. A. They kept me two days and rejected me as unfit for service with the Army. Again in 1916 I joined the R. G. A. I was only 37 days and was discharged as not being likely to become an efficient soldier on medical grounds. In 1917 I was under four medical boards. I was rejected each time and passed C. 3. In February 1918 I volunteered to come out in the M. T. I came out about three months ago and after being five days up the line I was sent to Hospital with Fits, Neurasthenia, and D. A. H. In June I was sent to my Base from No. 13 Convalescent Depot, was there about a week, and was sent back to Hospital again. I have some kind of attacks which take me suddenly. For years after them I lose my speech and also lose the use of my right side for some time. I cannot do much walking or lifting for I get pains in the right leg and across the back. I am also very short-winded."

The speaker wondered what the cost of that man had been to the state.

The speaker next considered what effect the convalescent depot may have on man-power and wastage. The treatment and methods adopted at convalescent depots, he said, vary enormously and it is an undoubted fact that if all depots were run on perfect lines the morale and mentality of the whole army would be affected.

It is absolutely essential that the right and only the right atmosphere should pervade the whole depot. *Sport and games* must be regarded as the most important part of the training. Under games should be included a system of physical training. *Boxing* above all must be encouraged to the greatest possible extent. This is the finest training for war these lads can have. Running contests have been especially effective as a means of keeping up the regimental spirit. Once men are made to participate in games and sports they soon begin to forget the sordid life of the trenches with all horrors and terrors which go to make up modern warfare. Besides games and sports there should be provided amusements of every kind, concerts, cinemas, bands, etc., as good food as possible and a varied diet scale; and there should be just enough discipline to remind the men that they are soldiers and that they have finished their Hospital treatment. The word "patient" should never be used in connection with a man in a convalescent depot. The more military the organization, the more quickly will results be achieved.

A convalescent depot is virtually a *training brigade* and should be organized into battalions, companies and platoons. The inter-company spirit and rivalry in games should be developed to the highest pitch by means of inter-company contests. To realize the benefits of a convalescent depot, one should see a convoy arrive, slouching, untidy, unsoldierly and disappointed men. Then one should see an evacuation party, smart, clean, well set up soldierly men going away in good spirits and with smiling faces though knowing full well what they are going back to — whistling shells!

The speaker believed that above all there should be an adequate staff of *permanent* workers at the depot. If convalescents were called on for too much of the routine work it greatly reduced the good effects of the rest. The speaker understood that the American arrangements had been planned on a smaller scale than the English, and he feared that this was a mistake.

The types of cases included under these Mild *P. U. O.'s* and very slight Wounds ought never to be sent to the convalescent depot. They could be dealt with better at a corps or army rest station.

